# Circulation of Coxsackievirus A10 and A6 in Hand-Foot-Mouth Disease in China, 2009–2011

**DOI:** 10.1371/journal.pone.0052073

**Published:** 2012-12-18

**Authors:** Qing-Bin Lu, Xiao-Ai Zhang, Ying Wo, Hong-Mei Xu, Xiu-Jun Li, Xian-Jun Wang, Shu-Jun Ding, Xiao-Dan Chen, Cui He, Li-Juan Liu, Hao Li, Hong Yang, Ting-Yu Li, Wei Liu, Wu-Chun Cao

**Affiliations:** 1 Department of Epidemiology and Health Statistics, Shandong University, Jinan, People’s Republic of China; 2 State Key Laboratory of Pathogen and Biosecurity, Beijing Institute of Microbiology and Epidemiology, Beijing, People’s Republic of China; 3 Children’s Hospital, Chongqing Medical University, Chongqing, People’s Republic of China; 4 Department of Infectious Disease Control, Shandong Provincial Disease Prevention and Control Center, Jinan, People’s Republic of China; Institut Pasteur of Shanghai, Chinese Academy of Sciences, China

## Abstract

Coxsackieviruses A10 (CV-A10) and A6 (CV-A6) have been associated with increasingly occurred sporadic hand-foot-mouth disease (HFMD) cases and outbreak events globally. However, our understanding of epidemiological and genetic characteristics of these new agents remains far from complete. This study was to explore the circulation of CV-A10 and CV-A6 in HFMD and their genetic characteristics in China. A hospital based surveillance was performed in three heavily inflicted regions with HFMD from March 2009 to August 2011. Feces samples were collected from children with clinical diagnosis of HFMD. The detection and genotyping of enteroviruses was performed by real-time PCR and sequencing of 5′UTR/VP1 regions. Phylogenetic analysis and selection pressure were performed based on the VP1 sequences. Logistic regression model was used to identify the effect of predominant enterovirus serotypes in causing severe HFMD. The results showed 92.0% of 1748 feces samples were detected positive for enterovirus, with the most frequently presented serotypes as EV-71 (944, 54.0%) and CV-A16 (451, 25.8%). CV-A10 and CV-A6 were detected as a sole pathogen in 82 (4.7%) and 44 (2.5%) cases, respectively. Infection with CV-A10 and EV-71 were independently associated with high risk of severe HFMD (OR = 2.66, 95% CI: 1.40–5.06; OR = 4.81, 95% CI: 3.07–7.53), when adjusted for age and sex. Phylogenetic analysis revealed that distinct geographic and temporal origins correlated with the gene clusters based on VP1 sequences. An overall *ω* value of the VP1 was 0.046 for CV-A10 and 0.047 for CV-A6, and no positively selected site was detected in VP1 of both CV-A10 and CV-A6, indicating that purifying selection shaped the evolution of CV-A10 and CV-A6. Our study demonstrates variety of enterovirus genotypes as viral pathogens in causing HFMD in China. CV-A10 and CV-A6 were co-circulating together with EV-71 and CV-A16 in recent years. CV-A10 infection might also be independently associated with severe HFMD.

## Introduction

Hand-foot-mouth disease (HFMD) is a common disease characterized with fever, sore throat, general malaise and vesicular eruptions on hand, feet, oral mucosa and tongue. Since 1997, several large epidemics of HFMD have been reported in the Asia-Pacific region, especially in Southeast Asia. Although HFMD is classically a mild disease, outbreaks in Asia have been associated with a high incidence of fatal cardiopulmonary and neurologic complications. HFMD has now become a notifiable disease in many countries [1].

Historically, outbreaks of HFMD were mainly caused by two types of enterovirus A species, enterovirus 71 (EV-71) and coxsackievirus A16 (CV-A16), with differing ratios. In recent years, coxsackieviruses A10 (CV-A10) and A6 (CV-A6), in addition to EV-71 and CV-A16, have been associated with increasingly occurred sporadic HFMD cases and outbreak events globally [2], [3], [4], [5], [6], [7], [8], [9], [10], [11]. In the largest outbreak of HFMD in Singapore in 2008, the most prevalent virus serotypes were demonstrated to be CV-A6 and CV-A10, accounting for 35.3% of the detected cases [3]. Large outbreaks of HFMD were reported to be caused by the co-circulating of CV-A10 and CV-A6 in Finland [11]. A sentinel surveillance study performed in France found CV-A10 and CV-A6 to be the most predominant HEV serotypes, which were also responsible for the outbreak events in 2010 [12]. One study performed in India in 2012 documented CV-A16 and CV-A6 as major, while CV-A10 and EV-71 as rare viral pathogens of HFMD [10]. One onychomadesis outbreak that occurred in 2008 in Spain was demonstrated to be associated with an outbreak of HFMD primarily caused by CV-A10 [5]. During 2008, an outbreak of HFMD with onychomadesis as a common feature occurred in Finland was identified to be caused by CV-A6 [2]. CV-A6, as the main serotype, also caused outbreaks of HFMD in Taiwan, 2010 [8] and in Japan, 2010 [9]. All of these previous studies provided strong evidence of CV-A6 and CV-A10 infections as new and important causes of HFMD, thus highlighting the necessity of comprehensive surveillance of all HEVs circulation in HFMD epidemics.

In China, there have been large outbreaks of HFMD every year in the past 3 years, each involving more than 500,000 cases, with an increasing number of neurologic symptoms and deaths reported. HFMD has become an important public health concern in China mainland. According to previous surveillance, EV-71 and CV-A16 have co-circulated as two most frequent HEV types in causing repeated HFMD outbreak in different areas [13], [14], [15], [16]. A few studies have also attempted to clarify the roles of other enteroviruses types, and identified only minor roles of CV-A10 and CV-A6 in China [15], [17]. Since most of the studies were performed before 2009 based on a small sample size, we have herein broadened these analyses to include more regions and over a longer time span in order to provide a more comprehensive overview of the viral pathogens of HFMD, with an emphasis to explore the prevalence of CV-A10 and CV-A6 in causing HFMD, as well as their epidemiological, clinical and genetic characteristics.

## Materials and Methods

### Sample Collection

The sentinel surveillance was performed from March 2009 to August 2011. The children suffering HFMD in three pediatric hospitals, which were set as the sentinel sites under national surveillance program for HFMD in Chongqing municipality (southwest China), Henan (central China) and Shandong province (east China) were recruited into the study. The patients were identified according to the diagnostic criteria defined by Ministry of Health (http://www.moh.gov.cn/publicfiles/business/htmlfiles/mohyzs/s3586/201004/46884.htm). Briefly, children were clinically diagnosed as HFMD if they had fever and onset of at least one of the following features: maculopapular of vesicular rash on the palms and/or soles and vesicles or ulcers in the mouth. Children with serious complications, including encephalitis, meningitis, acute flaccid paralysis, cardiorespiratory failure or death, were considered as severe HFMD. By the standard criteria in different hospitals, meningitis was defined as pleocytosis in cerebrospinal fluid analysis, encephalitis was characterized by the presence of altered level of consciousness, personality changes, or hallucinations. Children diagnosed with HFMD, but without above mentioned serious complications, were classified as mild HFMD.

Medical records of the patients were reviewed by physicians to collect the demographic data, the clinical symptoms and signs, laboratory findings, clinical diagnoses, and outcomes. Written informed consents were acquired from parents or guardians of all participants. The study was approved by the Ethics Review committee of Chongqing Medical University, Jining Infectious hospital and Armed Police Henan Hospital.

### Detection and Genotyping of HEV

Feces samples were collected and screened for HEV. Briefly, RNA were extracted from each specimen by using QIAamp® MinElute Virus Spin Kits (QIAGEN, Hilden, Germany) and the cDNA sample was synthesized by using SuperScript® III First-Strand Synthesis System for Reverse Transcription Polymerase Chain Reaction (RT-PCR) (Invitrogen, America).

The detection of HEV and further classification of EV-71 and CV-A16 for HEV-positive samples were performed by real-time PCR using previously described primers, respectively [18]. To further identify the HEV serotypes other than EV-71 and CA-A16, RT-PCR specific for a partial sequence of the 5′UTR was performed for other HEV-positive samples by using previously reported primers [19]. The amplicons were subject for sequencing and BLAST.

### Sequence Analysis of CV-A10 and CV-A6

The VP1 sequences for CV-A10 and CV-A6 positive samples were amplified by semi-nested PCR using the previously described primers 222, 224, 486, 488, AN88 and AN89 [16], [20].The genomic sequences were assembled using Lasergene’s DNA SeqMan software (version 7.1.0, DNA Star Inc. Madison, WI, USA). The sequences obtained from the study were submitted to NCBI and the GenBank Accession Numbers were JX947652–JX947838. All comparison alignments were performed and phylogenetic tree was constructed by neighbor-joining method with 1000 bootstrap replications using CLC genomics Workbench (version 5.1, developed by CLC bio). Similarities between strains were calculated by using BioEdit (version 7.13, www.mbio.ncsu.eud/bioedit/bioedit.html).

The value of ω and the individual site specific selection pressure were measured by using the single likelihood ancestor counting (SLAC), fixed effects likelihood (FEL) and random effects likelihood (REL) methods implemented in the Hypothesis testing using Phylogenies (HYPHY) package. Single breakpoint recombination (SBP) test with both AIC and BIC was conducted in this study. The overall ω value and 95% confidence interval (CI) were estimated based on NJ trees under the TrN93 substitution model. Selective pressure was defined as follows: *ω* = 1 indicates neutral evolution; *ω*<1 indicates purifying or negative selection; *ω*>1 indicates positive selection. *P* values less than 0.05 and Bayes factors larger than 20 were used as thresholds for strong evidence of selection in SLAC, FEL and REL, respectively. The results were confirmed by the codon-substitution model (PAML package 4.6, available at http://abacus.gene.ucl.ac.uk/software/paml.html).

### Statistical Analysis

Descriptive statistics were performed, with continuous variables summarized as median and range, and categorical variables summarized as frequencies and proportions. Chi-square test/Fisher exact or non-parametric test was used to see the difference between two groups. Logistic regression model was used to identify the association between severe HFMD and the viral pathogens after adjusting for age and sex. All the statistical analyses were conducted by SAS 9.13 (SAS Institute, Cary, North Carolina).

## Results

**Figure 1:**
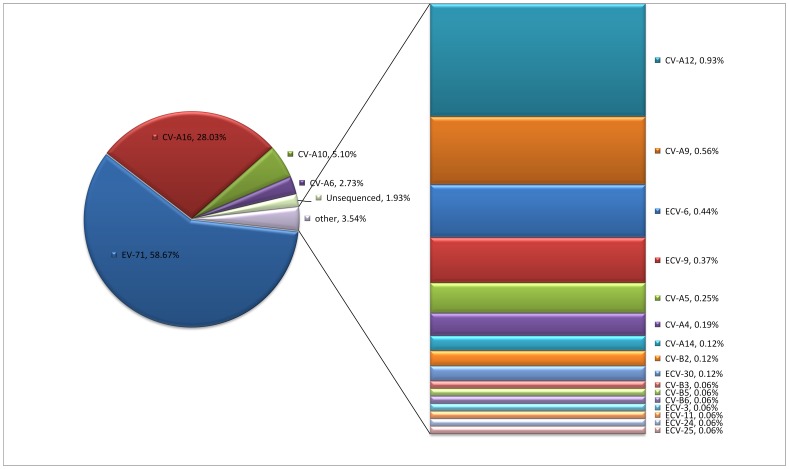
The enterovirus type identified in hand-foot-mouth diseases patients during Mar 2009 to Aug 2011, China. EV, enterovirus; CV, coxsackievirus; ECV, echovirus.

**Figure 2:**
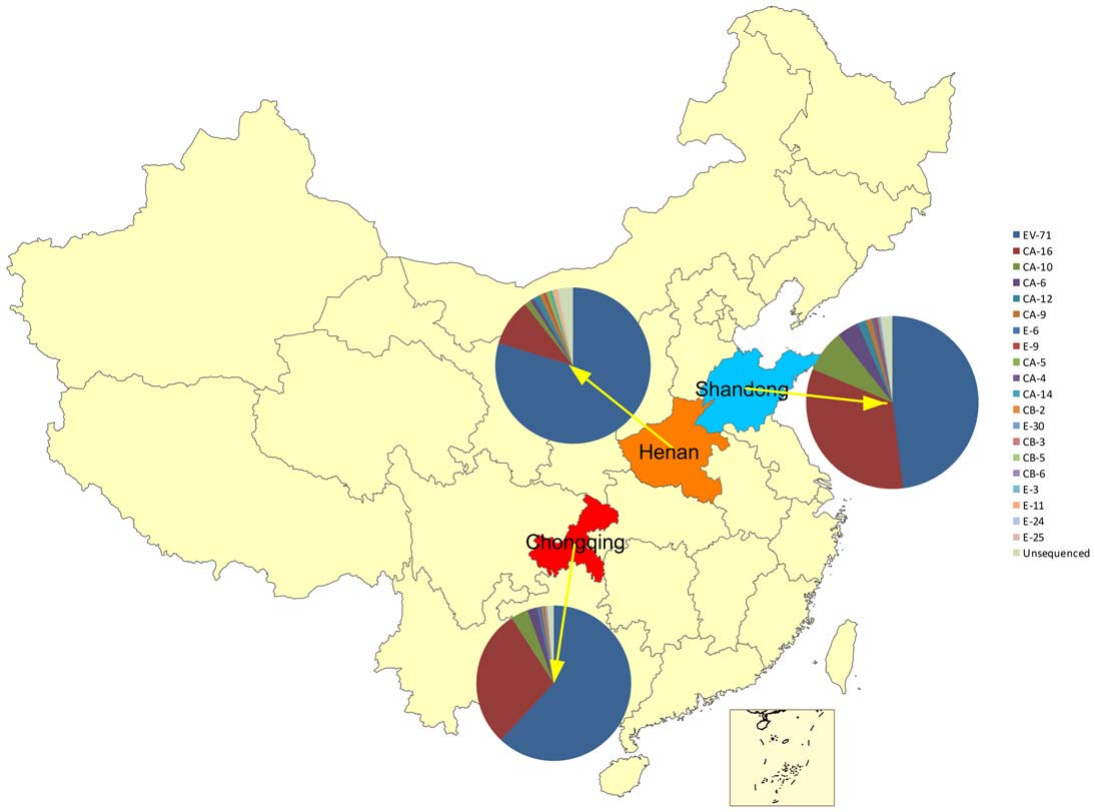
The detailed distribution of enterovirus types in each province. EV, enterovirus; CV, coxsackievirus; ECV, echovirus.

From March 2009 to August 2011, a total of 1748 children were recruited in the study with age ranging from 1 month to 15 years (median: 2 years) and 1125 (64.4%) were male. Altogether 92.0% of the 1748 feces samples were detected to be positive for HEV, with the most frequently presented serotypes as EV-71 (944, 54.0%) and CV-A16 (451, 25.8%). In addition, CV-A10 and CV-A6 were detected as a sole pathogen in 82 (4.7%) and 44 (2.5%) cases, respectively (Figure 1). The detailed distribution of enterovirus types in each province were shown in Figure 2.

The sex and age distribution of HFMD cases studied is shown in Table 1. The median age of CV-A10 positive cases was 25 months (range 7 months to 85 months), which was close to the median age of all HEV positive cases (24 months, range 1 month to 14 years). The median age of CV-A6 positive cases was 18 months, which was lower than the patients with other infection groups. No significant differences were identified among different enterovirus serotypes in terms of gender distribution. Respiratory and digestive syndromes were evenly distributed among each group, while syndrome of cyclic system and nervous system were significantly overrepresented among the patients infected with EV-71/CV-A10 than in CV-A16/CV-A6.

**Table 1:** The demographic and clinical characteristics of patients with different enteroviruses infection.

Characteristics	CV-A10 (n = 82)	CV-A6 (n = 44)	EV-71 (n = 944)	CV-A16 (n = 451)	*P*
Age, months (median, range)	25(7–85)	18(10–59)	24(2–168)	24(1–178)	0.128
Sex, male (%)	54(65.9)	27(61.4)	598(63.4)	311(69.0)	0.211
Clinical manifestations					
Respiratory system	17(21.5)	7(16.7)	198(25.6)	94(21.9)	0.281
Digestive system	8(10.1)	1(2.4)	42(5.5)	21(4.9)	0.224
Cyclic system	11(13.9)	4(9.5)	125(16.3)	32(7.5)	<0.001
Nervous system	24(29.3)	8(18.2)	497(52.4)	101(22.4)	<0.001
Outcome					<0.001
Mild	52(63.4)	36(81.8)	438(46.4)	335(74.3)	
Severe	30(36.6)	8(18.2)	506(53.6)	116(25.7)	

Note: respiratory system syndromes were defined as the presence of at least one of the followings: cough, bronchitis or other upper respiratory tract disease, or pneumonia; digestive system syndromes were defined as the presence of at least one of the followings: diarrhea or vomit; cyclic system syndromes were defined as the presence of at least one of the followings: myocarditis or cardiac damage; nervous syndromes were defined as the presence of at least one of the followings: meningitis, encephalitis, brain myelitis, coma, acute flaccid paralysis or seizures.

**Figure 3:**
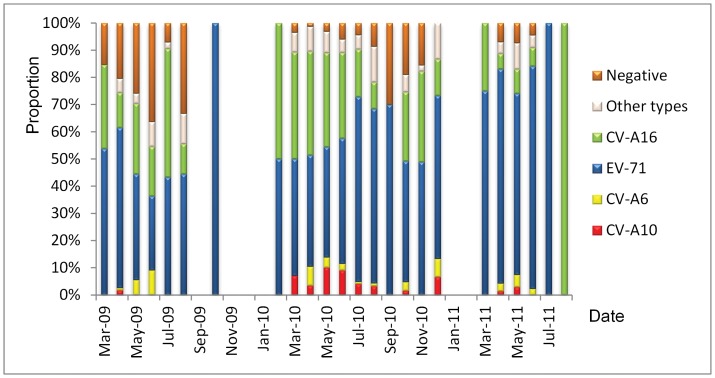
The temporal distribution of HEV types in hand-foot-mouth diseases patients during March 2009 to August 2011, China.

Similar to EV-71 and CV-A16, the prevalent season for CV-A10 and CV-A6 was warm season from April to August, with the proportion of CV-A10 and CV-A6 among the total enterovirus types attaining peak both in May for CV-A10 (51.2%) and CV-A6 (41.2%), respectively (Figure 3 and Table 2). When compared geographically, the proportions of CV-A10 and CV-A6 were significantly higher in Shandong Province than in other two provinces. When compared temporally, the proportions of CA-10, EV-71 and CV-A16 were different among the three years (Table 2).

**Table 2:** The composition of the enteroviruses types in the children with HFMD in each sampling month and region.

Date	CV-A10			CV-A6		EV-71			CV-A16			Other types			Negative	Total	
	n	%		n	%	n	%		n	%		n	%		n	%	n
Mar-09	0	0.0		0	0.0	7	53.8		4	30.8		0	0.0		2	15.4	13
Apr-09	2	1.7		1	0.9	69	59.5		15	12.9		6	5.2		24	20.7	116
May-09	0	0.0		3	5.6	21	38.9		14	25.9		2	3.7		14	25.9	54
Jun-09	0	0.0		1	9.1	3	27.3		2	18.2		1	9.1		4	36.4	11
Jul-09	0	0.0		0	0.0	55	43.0		60	46.9		3	2.3		9	7.0	128
Aug-09	0	0.0		0	0.0	4	44.4		1	11.1		1	11.1		3	33.3	9
Oct-09	0	0.0		0	0.0	1	100.0		0	0.0		0	0.0		0	0.0	1
Feb-10	0	0.0		0	0.0	1	50.0		1	50.0		0	0.0		0	0.0	2
Mar-10	2	7.1		0	0.0	12	42.9		11	39.3		2	7.1		1	3.6	28
Apr-10	5	3.7		10	7.5	59	44.0		55	41.0		13	9.7		2	1.5	134
May-10	38	10.4		14	3.8	152	41.5		130	35.5		29	7.9		12	3.3	366
Jun-10	15	9.3		4	2.5	76	47.2		52	32.3		8	5.0		10	6.2	161
Jul-10	10	4.0		2	0.8	168	68.0		43	17.4		13	5.3		11	4.5	247
Aug-10	3	3.3		1	1.1	59	64.1		9	9.8		12	13.0		8	8.7	92
Sep-10	0	0.0		0	0.0	7	70.0		0	0.0		0	0.0		3	30.0	10
Oct-10	1	1.6		2	3.1	28	43.8		16	25.0		4	6.3		12	18.8	64
Nov-10	0	0.0		0	0.0	22	46.8		15	31.9		1	2.1		7	14.9	47
Dec-10	1	7.1		1	7.1	9	64.3		2	14.3		2	14.3		0	0.0	14
Mar-11	0	0.0		0	0.0	3	75.0		1	25.0		0	0.0		0	0.0	4
Apr-11	1	1.4		2	2.9	56	81.2		4	5.8		3	4.3		5	7.2	69
May-11	4	3.1		6	4.7	90	69.8		12	9.3		13	10.1		10	7.8	129
Jun-11	0	0.0		1	2.3	36	83.7		3	7.0		2	4.7		2	4.7	43
Jul-11	0	0.0		0	0.0	6	100.0		0	0.0		0	0.0		0	0.0	6
Aug-11	0	0.0		0	0.0	0	0.0		1	100.0		0	0.0		0	0.0	1
Year																	
2009	2	0.6		5	1.5	160	48.2		96	28.9		13	3.9		56	16.9	332
2010	75	6.4		34	2.9	593	50.9		334	28.7		84	7.2		66	5.7	1165
2011	5	2.0		9	3.6	191	75.8		21	8.3		18	7.1		17	6.7	252
*P*	<0.001			0.261		<0.001			<0.001			0.094			<0.001		
Region																	
Chongqing	26		3.4	15	1.9	446		57.9	208		27.0	25		3.2	50	6.5	770
Shandong	53		7.2	27	3.6	318		42.9	221		29.8	44		5.9	78	10.5	741
Henan	3		1.3	2	0.8	180		75.9	22		9.3	19		8.0	11	4.6	237
*P*	<0.001			0.023		<0.001			<0.001			0.005			0.002		

There were 725 (41.5%) patients diagnosed as severe HFMD. The risk factors for severe HFMD were evaluated based on the multivariate logistic regression analysis (Table 3). When adjusted for the effect from age and gender, CV-A10 and EV-71 were independently associated with higher risk of severe HFMD (OR = 2.66, 95%CI: 1.40–5.06; OR = 4.81, 95%CI: 3.07–7.53).

**Table 3:** Logistic regression analysis on the effect of predominant enterovirus serotypes in severe HFMD.

Variable	Unadjusted			Adjusted		
	OR	95%CI	*P*	OR	95%CI	*P*
Age, months						
≤12	4.26	2.77–6.54	<0.001	4.25	2.72–6.64	<0.001
12∼24	2.05	1.41–2.99	<0.001	2.08	1.41–3.07	<0.001
24∼36	1.78	1.20–2.65	0.004	1.85	1.22–2.79	0.004
36∼48	2.22	1.45–3.39	<0.001	2.16	1.39–3.36	<0.001
>48	1.0			1.0		
Sex, male/female	0.87	0.71–1.06	0.168	0.94	0.75–1.17	0.556
Enterovirus						
EV-71	4.58	2.97–7.07	<0.001	4.81	3.07–7.53	<0.001
CV-A16	1.37	0.86–2.19	0.182	1.51	0.94–2.45	0.092
CV-A10	2.29	1.24–4.22	0.008	2.66	1.40–5.06	0.003
CV-A6	0.88	0.37–2.11	0.776	1.20	0.48–3.02	0.700

Identity analyses based on 5′UTR sequences revealed both CV-A10 and CV-A6 were highly conserved within the same serotype.

**Figure 4:**
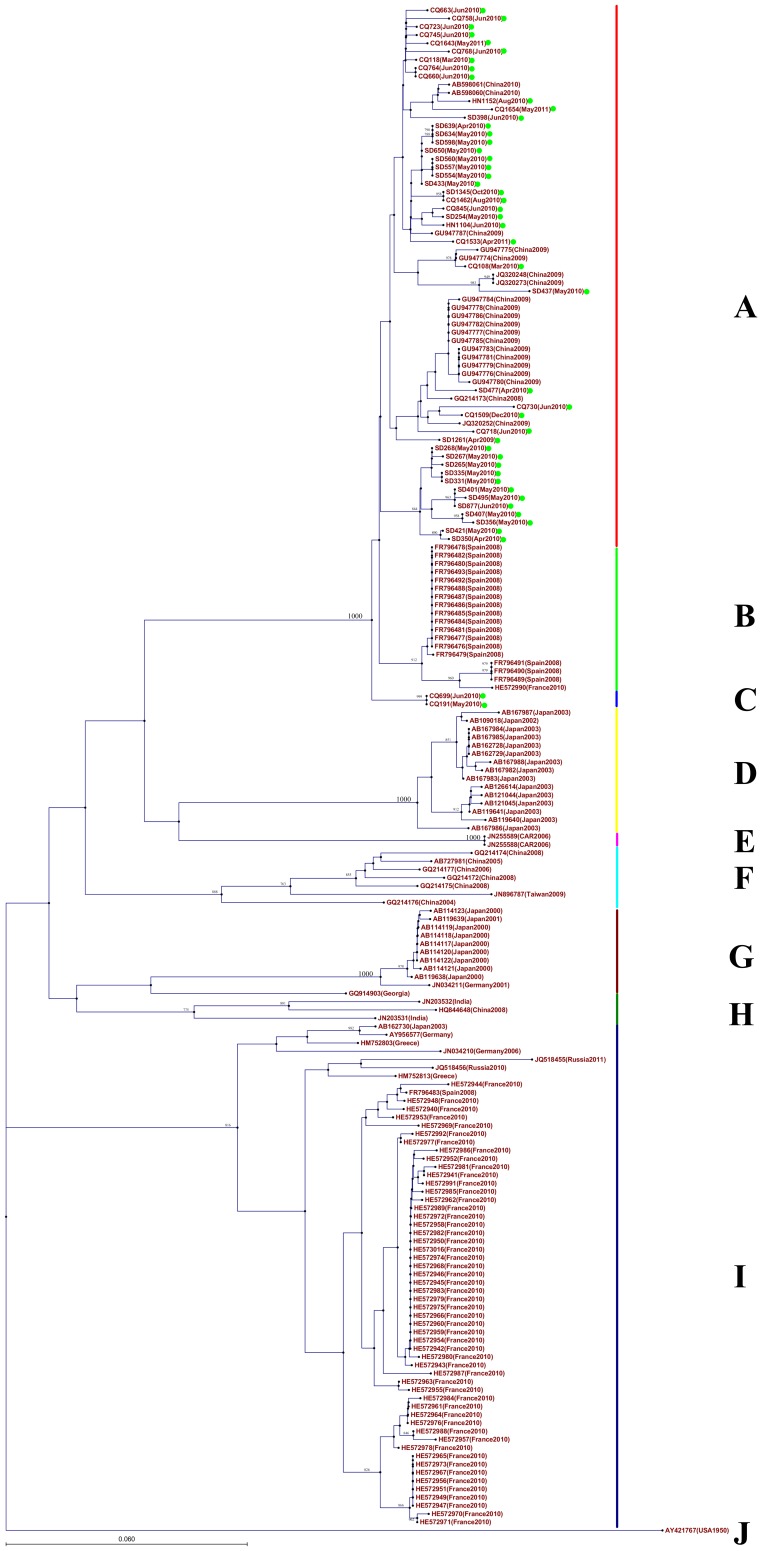
Phylogenetic trees were constructed from the VP1 nucleotide sequences of CV-A10 using neighbor-joining method with 1000 bootstrap by CLC genomics workbench. The tree was based on the 379 bp VP1 nucleotide sequences of CV-A10 (nt 2630–3008, responding to AY421767). The strains labeled with green dot were obtained in our study.

**Figure 5:**
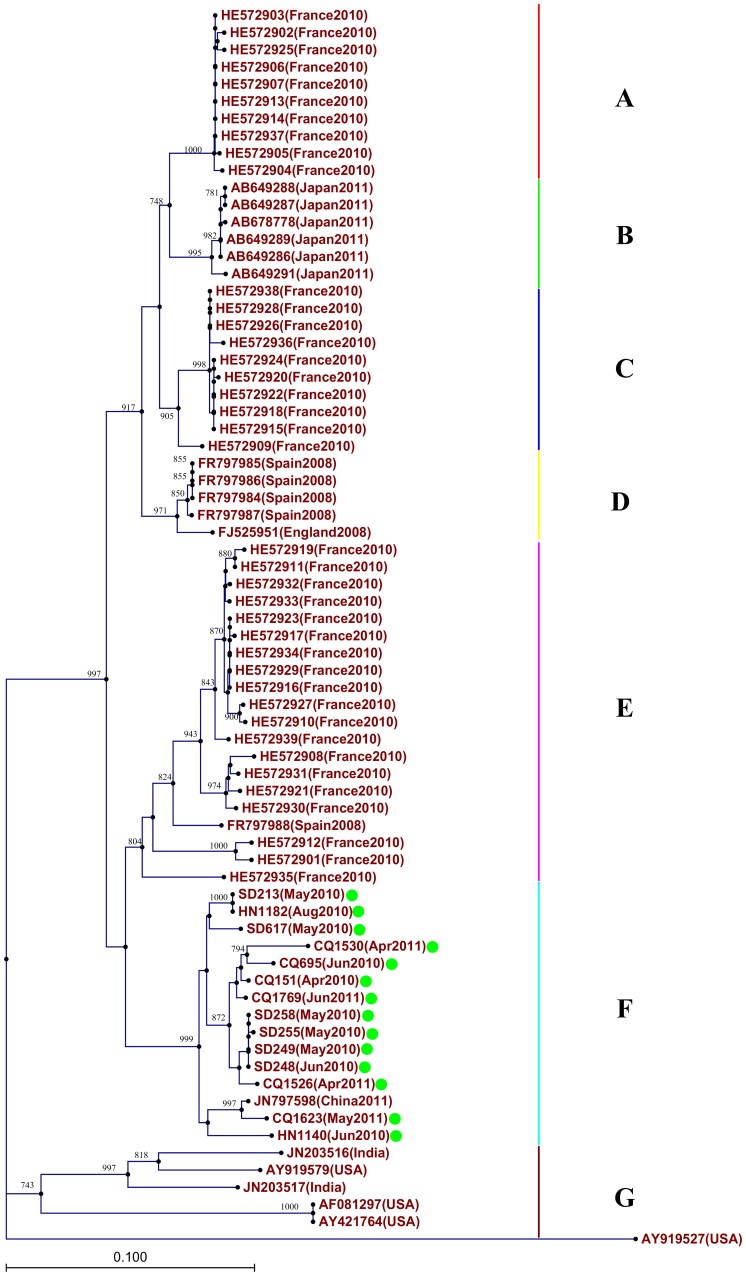
Phylogenetic trees were constructed from the VP1 nucleotide sequences of CV-A6 using neighbor-joining method with 1000 bootstrap by CLC genomics workbench. The tree was based on the 552 bp VP1 nucleotide sequences of CV-A6 (nt 2441–2992, responding to AY421764). The strains labeled with green dot were obtained in our study.

Two phylogenetic trees were constructed by the VP1 nucleotide sequences of CV-A10 and CV-A6 from the present study and those downloaded from GenBank, respectively (Figure 4 and Figure 5). The CV-A10 sequences were assigned to ten clusters (A-J) with clear geographical and temporal specific distributions (Figure 4): All Chinese CV-A10 strains were segregated into four distinct branches: Cluster A comprising the strains in this study and other strains from Yunnan, Fujian, and Shandong, China during 2009–2011; Cluster C including strain detected exclusively from Chongqing in 2010; Cluster F with strains from Yunnan, Shandong and Taiwan, China before 2009; Cluster H with one stain from Yunan,2008, together with the strains from India. The temporal characteristics were also obvious, with the strains of same geographic origin whereas different sampling time were clustered separately, for example, the Shandong strains isolated in 2010/2011 and 2008 were classified into different clusters (A and F, respectively) Cluster B, includes the 2008 strains from Spain and one 2010 strain from France; Cluster D includes the 2003 strain from Japan; Cluster E includes two 2006 strains from Central Africa Republic; Cluster G mainly includes the 2000/2001 strains from Japan; Cluster I includes the European strains; Cluster J includes one strain from USA. For CV-A6, 7 clusters (A-G) were formed with obvious geographical distribution pattern (Figure 5): Cluster A, C and E include the strains from France; Cluster B includes the strains from Japan; Cluster D includes the strains from Spain and one from England; Cluster F includes the strains from China; Cluster G includes the strains from USA and India.

Global selective pressure was examined on the determinant encoding VP1 for CV-A10 (564 bp, nt 1–564 of VP1) and for CV-A6 (552 bp, nt 1–552 of VP1). Before making the inference of positive selection, recombination should be taken into account to avoid biases. The result of SBP test showed that there was no evidence for recombination in VP1 gene of CV-A10/CV-A6. An overall *ω* value of the VP1 was 0.046 (95% CI: 0.038–0.056) for CV-A10 and 0.047 (95% CI: 0.039–0.057) for CV-A6, indicating that the amino acids in the antigenic determinant were under purifying selection. Site-by-site analyses were performed to determine whether any specific site or residue of the VP1 region evolved under positive selection. No positively selected site was detected from CV-A10 dataset through the methods of SLAC, FEL and REL. Similarly, no positively selected site was detected for CV-A6, and although only one positively selected site (codon site: 12) were detected by SLAC method (*dN*-*dS* = 24.45, *P*<0.001), this site was not detected by FEL or REL methods. There were no positively selected site to detect by the codon-substitution model using PAML package 4.6.

## Discussion

HFMD is a common disease among children, especially under 5 years. HFMD is benign and self-limiting, but severe HFMD with complications are also developed. Numerous major epidemics of HFMD have occurred in eastern and southeastern Asian countries and regions in the past decade, with EV-71 as the most commonly responsible enterovirus type [21], [22], [23], [24]. However, other types, especially CV-A10 and CV-A6, began to co-circulate with increasingly frequencies in recent years, turning into equally common causes of HFMD as CV-16 and EV-71in certain regions [12], [21], [25]. In mainland China, the surveillance on HFMD was mostly focused on EV-71 and CV-A16, therefore, information on the pathogenic role of other enteroviruses, their geographic distribution and epidemiological profiles are also still lacking. Our study, based on the latest surveillance in three heavily inflicted regions with HFMD, demonstrated that the previously infrequently detected CV-A10 and CV-A6, are becoming important HFMD pathogens, although the major HFMD pathogens remained to be EV-71 and CV-A16. Therefore, virological surveillance to detect more serotypes concurrently is necessary and physicians should be aware of these emerging pathogens.

Previous studies demonstrated that EV-71 is more likely to cause serious complications than other enterovirus types and usually leads to meningoencephalitis, pulmonary hemorrhage, circulation failure and death. One case-control study also revealed that EV-71 was significantly associated with an increased risk of severe HFMD (OR = 39.17, 95%CI: 9.80–156.52) [26]. In our study, CV-A10 was demonstrated to be associated with severe complications defined by the same criteria, although with a less effect than that of EV-71. Further studies should be intensified to isolate CV-A10 and investigate its genetic and pathogenic features for clinical management.

Our study identified distinct clusters of CV-A10 and CV-A6 strains that related to their geographic origins. Moreover, CV-A10 also displayed diverse genetic characteristics regarding their temporal sources. According to previous studies from Yang et al. and Wang et al., CV-A10 analyzed during 2008–2009 in Shandong and during 2009 in Beijing [16], [17] formed different clusters with strains from Japan, respectively, therefore displaying clear cut temporal distribution. Hu et al. found natural recombination is a frequent event in human enterovirus A evolution [27]. Based on the current analysis, no evidence of recombination was revealed for VP1 gene of CV-A10. Further evolutionary studies, representing much more geographic locations and genetic information, would help to improve our understanding of its evolutionary relationships. Due to the limited VP1 nucleotide sequences of CV-A6 submitted to GenBank, the temporal characteristic of CV-A6 cannot be inferred, which warrants further investigation in the future.

CV-A10 and CV-A6, both belonging to enterovirus A group, had the similar global *ω* and no positively selected sites were detected. Our study shows that purifying selection plays an important part in shaping the evolution of CV-A10 and CV-A6. This constrained mutation of the two genotypes can be explained by the limited size and the genetic architecture of the viral genome which is overlapping between structural and functional domains like other viruses [28], [29].

In summary, our study demonstrates variety of enterovirus genotypes in the pathogens of HFMD in China based on more than two-year surveillance. CV-A10 and CV-A6 were co-circulating with EV-71 and CV-A16 in recent three years. More wide regional surveillance is warranted to predict their potential in causing outbreak event, as reported from other countries. CV-A10 infection might also be associated with severe HFMD. Its diverse genetic characteristics, as well as distinct geographical distribution were also disclosed. Further genomic analysis and molecular epidemiological data of CV-A10 might help to track the spread of the virus across the country.
